# Supplementary material for: Perceptions of influenza and SARS-CoV-2 vaccination among health care personnel in Thailand, 2024
Source: PLoS One. 2025 Aug 14;20(8):e0329473. doi: 10.1371/journal.pone.0329473 (PMC12352830; doi:10.1371/journal.pone.0329473)
Supplement: S1 File — Survey instrument developed for this study. (PDF) [file pone.0329473.s001.pdf]

Annex 7 Questionnaire

ID ☐ ☐ ☐ ☐ ☐ ☐

Knowledge, Attitudes, Practices, and Perceptions (KAPP) Survey of Health Care Workers

Date of interview..

Name(s) of interviewer(s):

1. Name – Role.. (Interviewer/Notetaker)
2. Other

(If other) Specify name of interviewer: ....

LOCATION
☐ Thailand

Region
☐ 1 North ☐ 2 Central ☐ 3 South ☐ 4 Northeast

Rural or Urban
☐ 1 Rural ☐ 2 Urban

Health Facility Type
☐ 1 Provincial level hospital ☐ 2 District level hospital

Health Facility Name
☐ 1 Chiang Rai Prachanukroh Hospital
☐ 2 Phayao Hospital
☐ 3 Uthai Thani Hospital
☐ 4 Ratchaburi Hospital
☐ 5 Nakhon Phanom Hospital
☐ 6 Nong Khai Hospital
☐ 7 Surat Thani Hospital
☐ 8 Chumphon Khet Udomsak Hospital
☐ 9 Mae Sai Hospital
☐ 10 Dokkhamtai Hospital
☐ 11 Thap Than Hospital
☐ 12 Photharam Hospital
☐ 13 Phonsavan Hospital
☐ 14 Somdej Phra Yupparat Tha Bo Hospital
☐ 15 Tha Chang Hospital
☐ 16 Lang Suan Hospital

Are you able to get vaccinated with the influenza vaccine if you wanted to?
☐ 1 Yes
☐ 2 No
☐ 3 I don't know/Unsure

SECTION 1: General and Demographic Questions

[INTERVIEWER: Thank you for your time. I would like to begin by asking some general demographic questions.]

Instruction: Please answer the question by marking in the selected ☐ box.

General and Demographic	Answer options
A1. Gender	☐ 1 Male<br>☐ 2 Female<br>☐ 3 Prefer not to say
A2. Age (Years)	…………….year old
A3**.** How many years have you worked in health care?	…………….year
A4. What is your occupation?	☐ 1 Doctor<br>☐ 2 Assistant Doctor<br>☐ 3 Nurse<br>☐ 4 Assistant Nurse<br>☐ 5 Midwife<br>☐ 6 Dentist<br>☐ 7 Pharmacist<br>☐ 8 public health officer<br>☐ 9 emergency medical worker (EMT)/ Rescue<br>☐ 10 laboratory staff<br>☐ 11 patient assistant<br>☐ 12 Other, please specify............................
A5. In which specialty area(s), department(s) or unit(s) do you primarily work?	☐ 1 General / Internal Medicine<br>☐ 2 Adult Intensive Care Unit<br>☐ 3 Neonatal Intensive Care Unit<br>☐ 4 Emergency Department<br>☐ 5 Obstetrics / Gynecology<br>☐ 6 Pediatrics<br>☐ 7 Radiology<br>☐ 8 Surgery<br>☐ 9 Other, please specify: ....
A6. What type of patients do you generally treat?	☐ 1 Pregnant women<br>☐ 2 Children<br>☐ 3 Adults with chronic diseases<br>☐ 4 Adults with infectious diseases<br>☐ 5 Older adults (above 65)<br>☐ 6 Other, please specify: ....
A7 a. Has a doctor or another health worker told you that you have a long-term chronic health condition? This could include, for example, obesity, diabetes, lung disease, high blood pressure or another long-term condition.	☐ 1 Yes<br>☐ 2 No (Skip 7b)

A7 b. [IF YES] Which long-term health condition(s) do you have? (DO NOT READ RESPONSE CHOICES. MARK ALL THAT APPLY)	☐ 1 Obesity<br>☐ 2 Diabetes<br>☐ 3 Heart Disease<br>☐ 4 Lung Disease<br>☐ 5 Immunocompromised<br>☐ 6 Another Long-Term Condition (Specify):<br>..

SECTION 2: Influenza Disease & Influenza Vaccines
INTERVIEWER: Thank you for sharing your demographic information. I will now ask some questions related to influenza disease and vaccination.

I. General Knowledge and Perceptions

Instruction: Please answer the question by marking ✓ in the selected ☐ box.

I. General Knowledge and Perceptions	Answer options
B1. Have you ever made a clinical or laboratory-confirmed diagnosis of influenza in a patient?	☐ 1 Yes<br>☐ 2 No<br>☐ 3 I don't remember
B2. Have you ever treated a patient that developed a life-threatening complication (i.e., pneumonia or death) and required hospitalization because of influenza illness or suspected influenza illness?	☐ 1 Yes<br>☐ 2 No<br>☐ 3 I don't remember

Next, there are a set of statements about influenza severity of disease , safety, and effectiveness of influenza vaccines for several groups of people. Please indicate to what extent you agree or disagree with each statement for different groups of people.

B3. *Influenza disease severity:* "Every year, influenza can lead to hospitalizations, admissions to the intensive care unit (ICU) and/or death. For [SAY SPECIFIC GROUP OF PEOPLE BELOW], do you strongly agree, agree, disagree, or strongly disagree?"

Population group	Strongly Agree	Agree	Disagree	Strongly Disagree	Refuse to Answer
Health workers					
People in the general population					
Pregnant women					
A pregnant woman's fetus					
Persons with underlying chronic conditions					
Individuals aged ≥ 65 years					
Immunocompomised (from medication/treatments)					
Children <5 years					
School-aged children (5-16years old)					
Me / Self					

B4. *Influenza vaccine safety*   : "   Please say how safe you believe the influenza vaccine is for each of the groups below. Is the influenza vaccine very safe, safe, unsafe or very unsafe for [SAY SPECIFIC GROUP OF PEOPLE BELOW]"

**Population group**	**Strongly Agree**	**Agree**	**Disagree**	**Strongly Disagree**	**Refuse to Answer**
Health workers					
People in the general population					
Pregnant women					
A pregnant woman's fetus					
Persons with underlying chronic conditions					
Individuals aged ≥ 65 years					
Immunocompomised (from medication/treatments)					
Children <5 years					
School-aged children (5-16years old)					
Me / Self					

B5. *Influenza vaccine effectiveness:* "Getting the influenza vaccine can reduce the chances of becoming severely ill (i.e., requiring hospitalization) with influenza. For [SAY SPECIFIC GROUP OF PEOPLE BELOW], do you strongly agree, agree, disagree, or strongly disagree?"

Population group	**Strongly Agree**	**Agree**	**Disagree**	**Strongly Disagree**	**Refuse to Answer**
Health workers					
People in the general population					
Pregnant women					
A pregnant woman's fetus					
Persons with underlying chronic conditions					
Individuals aged ≥ 65 years					
Immunocompomised (from medication/treatments)					
Children <5 years					
School-aged children (5-16years old)					
Me / Self					

B6. **Can the influenza vaccine protect you from COVID-19 infection and illness?**
☐ 1 Yes   ☐ 2 No   ☐ 3 I don't know

II. Acceptance of Influenza Vaccines by Health Care Workers	Answer options
**B7. Do you remember the H1N1 influenza pandemic in 2009?**	☐ 1 Yes<br>☐ 2 No (skip to B9)
**B8. [IF YES] Did you get vaccinated during the 2009 influenza pandemic?**	☐ 1 Yes<br>☐ 2 No<br>☐ 3 I don't remember/I'm not sure
**B9. Did you ever receive an influenza vaccine prior to the onset of the COVID-19 pandemic?**	☐ 1 Yes<br>☐ 2 No<br>☐ 3 I don't remember/ I'm not sure (skip to B11)
**B10. Please explain why or why not:**<br>..<br>..	
**B11. Did you receive the influenza vaccine *in the last season* (2022)?**	☐ 1 Yes<br>☐ 2 No (skip to Q B11.2)<br>☐ 3 I don't remember/ I'm not sure (skip to B12)
**B11.1 If yes, what made you decide to receive the influenza vaccine (check all that apply)?**	☐ 1 I receive the vaccine every year<br>☐ 2 It was offered for free at my job (not mandatory)<br>☐ 3 It was mandatory at my job<br>☐ 4 To protect my patients<br>☐ 5 To protect myself from infection<br>☐ 6 To protect my family<br>☐ 7 It was easy/offered at my workplace<br>☐ 8 It was free<br>☐ 9 It is recommended by my Ministry of Health or my organization<br>☐ 10 Other reason (please explain)<br>..
**B11.2 If no, why did you decide not to be vaccinated (check all that apply)?**	☐ 1 The vaccine is not available to health workers in my country<br>☐ 2 I do not believe it will protect me from infection<br>☐ 3 I did not want to pay for it (not free at work)<br>☐ 4 I did not have time to go get vaccinated<br>☐ 5 I do not believe I am susceptible to influenza illness<br>☐ 6 I do not believe in vaccinations<br>☐ 7 I do not believe the influenza vaccine is safe<br>☐ 8 It is not recommended by my Ministry of Health or my organization<br>☐ 9 I prefer natural immunity<br>☐ 10 Vaccination is inconvenient There is difficulty in receiving service.

	☐ 11 A small number of vaccines, not enough<br>☐ 12 Vaccines have side effects or adverse reactions.<br>☐ 13 Vaccines make me sick<br>☐ 14 Don't like vaccinations, afraid of getting hurt<br>☐ 15 Other (please explain)<br>...
**B12. If influenza vaccine was recommended for health care workers and offered to you for free, would you get vaccinated?**	☐ 1 Yes<br>☐ 2 No (Skip to B14)<br>☐ 3 I don't know
**B13. What is the most important (top) reason to ACCEPT to take the influenza vaccine for yourself? (Skip to B15)**	....<br>...<br>...
**B14. What is the most important (top) reason to REFUSE to take the influenza vaccine for yourself?**	....<br>...<br>...

III. Recommendation of Influenza Vaccines by Health Care Workers

III. Recommendation of Influenza Vaccines by Health Care Workers	Answer options
**B15. If influenza vaccine was available for your patients, would you vaccinate your patients or recommend they receive the vaccine?**	☐ 1 Yes<br>☐ 2 No (Skip to B18)

B16. If yes, who are the top three groups of people you would vaccinate or would recommend they receive the vaccine against influenza [INTERVIEWER: DO NOT READ LIST ALOUD].

	Group 1	Group 2	Group 3
☐1 Healthy adults	1. ☐	1. ☐	1. ☐
☐2 Older adults (65 and older)	2. ☐	2. ☐	2. ☐
☐3 School age children (5-16 years old)	3. ☐	3. ☐	3. ☐
☐4 Children younger than 5 year	4. ☐	4. ☐	4. ☐
☐5 Women of childbearing age	5. ☐	5. ☐	5. ☐
☐6 Pregnant women	6. ☐	6. ☐	6. ☐
☐7 Health care workers	7. ☐	7. ☐	7. ☐
☐8 People with HIV/AIDS	8. ☐	8. ☐	8. ☐
☐9 People with Tuberculosis	9. ☐	9. ☐	9. ☐
☐10 Immunocompromised	10. ☐	10. ☐	10. ☐
☐11 Persons with underlying chronic conditions	11. ☐	11. ☐	11. ☐
☐12 I don't know	12. ☐	12. ☐	12. ☐

**B17. [If Yes to B15] What is the most important (top) reason that would make you RECOMMEND the influenza vaccine? (Skip to B19)**	☐ 1. To protect my patients<br>☐ 2. To protect my family<br>☐ 3. It is easy/offered at my workplace<br>☐ 4. It is free<br>☐ 5. It is recommended by my Ministry of Health or my organization<br>☐ 6. Other reason (please explain)<br>…………………………………………………………
**B18. [If No to 16], What is the most important (top) reason that would make you NOT RECOMMEND the influenza vaccine?**	....<br>..
**B19. Have you encountered any resistance or hesitancy from patients when recommending influenza vaccine? [in ODK, this should be asked to all]**	☐ 1 Yes<br>☐ 2 No (Skip to section 3)
**B19.1. If yes, what reason(s) do you hear for the resistance or hesitancy to receive the influenza vaccine?** (Select all that apply)	☐ 1 Access<br>☐ 1) Availability<br>☐ 2) Location of delivery site<br>☐ 3) Transportation<br>☐ 2 Time<br>☐ 1) Travel time to clinic<br>☐ 2) Waiting time (e.g., in line for vaccination)<br>☐ 3 Vaccine cost<br>☐ 4 Visit cost/other cost<br>☐ 5 Patient fear of safety<br>☐ 6 Other (please explain) ....<br>...

SECTION 3: COVID-19 Disease & COVID-19 Vaccines

INTERVIEWER: Thank you for sharing your thoughts on influenza disease and vaccination. I will now ask some questions related to COVID-19 disease and vaccination.

I. General Knowledge and Attitudes

I. General Knowledge and Attitudes	Answer options
**C1. Have you ever made a clinical or laboratory-confirmed diagnosis of COVID-19 in a patient?**	☐ 1 Yes<br>☐ 2 No<br>☐ 3 I don't remember
**C2. Have you ever treated a patient that developed a life-threatening complication (such as, pneumonia or death) and required hospitalization because of COVID-19 disease or suspected COVID-19 disease?**	☐ 1 Yes<br>☐ 2 No<br>☐ 3 I don't remember

Next, there are a set of statements about COVID-19 disease severity, safety, and effectiveness of influenza vaccines. Please indicate to what extent you agree or disagree with each statement.

C3. COVID-19 *disease severity*. "COVID-19 can cause serious illness, requiring hospitalization, long term care or death in unvaccinated [SAY NAME OF GROUP BELOW]. Do you strongly agree, agree, disagree, or strongly disagree?

Population group	Strongly Agree	Agree	Disagree	Strongly Disagree	Refuse to Answer
Health workers					
People in the general population					
Pregnant women					
A pregnant woman's fetus					
Persons with underlying chronic conditions					
Individuals aged ≥ 65 years					
Immunocompomised (from medication/treatments)					
Children <5 years					
School-aged children (5-16years old)					
Me / Self					

C4. *COVID-19 vaccination safety*. How safe do you believe COVID-19 vaccines are for [SAY NAME OF GROUP BELOW]? Are they very safe, safe, unsafe, or very unsafe?

Population group	Very Safe	Safe	Unsafe	Very Unsafe	Refuse to Answer
Health workers					
People in the general population					
Pregnant women					
A pregnant woman's fetus					

Persons with underlying chronic conditions					
Individuals aged ≥ 65 years					
Immunocompomised (from medication/treatments)					
Children <5 years					
School-aged children (5-16years old)					
Me / Self					

C5. ***COVID-19 vaccine effectiveness*****. Getting the COVID-19 vaccine can greatly reduce the chances of becoming severely ill (i.e., requiring hospitalization) with COVID-19 for [SAY NAME OF GROUP BELOW]. Do you strongly agree, agree, disagree, or strongly disagree?**

**Population**	**Strongly Agree**	**Agree**	**Disagree**	**Strongly Disagree**	**Refuse to Answer**
Health workers					
People in the general population					
Pregnant women					
A pregnant woman's fetus					
Persons with underlying chronic conditions					
Individuals aged ≥ 65 years					
Immunocompomised (from medication/treatments)					
Children <5 years					
School-aged children (5-16years old)					
Me / Self					

II. Acceptance of COVID-19 Vaccines by Health Care Workers

**ll. Acceptance of COVID-19 Vaccines by Health Care Workers**	**Answer options**
**C6. Have you received a COVID-19 vaccine since the vaccines were introduced in your country?**	☐ 1 Yes (skip to C7)<br>☐ 2 No (skip to C11)<br>☐ 3 I don't remember (skip to C11)
**C7. [IF YES TO 6] If your vaccine required more than one dose, did you complete your primary series/receive all your required doses?**	☐ 1 Yes (skip to C9)<br>☐ 2 No (skip to C8)<br>☐ 3 I don't know (skip to C11)

**C8. If you received only one dose of a multi-dose series, please explain why you did not receive additional doses:**<br>..<br>..<br>..	
**C9. Have you had one or more booster doses since you completed your primary series?**	☐ 1 Yes<br>☐ 2 No (Skip to C9.2)<br>☐ 3 I don't know (Skip to C10)
**C9.1. If yes, how many booster doses have you received?**	☐ 1 to receive..................... dose<br>☐ 2 I don't remember
**C9.2. If no, why not?**<br>..<br>..<br>..	
**C10. The World Health Organization (WHO) currently recommends continued vaccination of front-line health workers with COVID-19 vaccines. If such a recommendation exists in your country, will you continue to receive booster doses?**	☐ 1 Yes<br>☐ 2 No<br>☐ 3 I don't know
**C11. If COVID-19 vaccine becomes an annually recommended vaccine for health workers, like the seasonal influenza vaccine, will you be vaccinated every year with COVID-19 vaccine?**	☐ 1 Yes<br>☐ 2 No (Skip to C13)<br>☐ 3 I don't know (Skip to C14)
**C12. What is the most important (top) reason to ACCEPT to receive COVID-19 boosters or receive the COVID-19 vaccine annually for yourself?**	☐ 1 It was offered for free at my job (not mandatory)<br>☐ 2 It was mandatory at my job<br>☐ 3 To protect my patients<br>☐ 4 To protect myself from infection<br>☐ 5 To protect my family<br>☐ 6 It was easy/offered at my workplace<br>☐ 7 It was free<br>☐ 8 It is recommended by my Ministry of Health or my organization<br>☐ 9 Other reason (please explain)<br>……………….……………....…………
**C13. What is the most important (top) reason to REFUSE to take the COVID-19 boosters or receive the COVID-19 vaccine annually for yourself?**	☐ 1 The vaccine is not available to health workers in my country<br>☐ 2 do not believe it will protect me from infection<br>☐ 3 did not want to pay for it (not free at work)<br>☐ 4 did not have time to go get vaccinated<br>☐ 5 do not believe I am susceptible to influenza illness<br>☐ 6 do not believe in vaccinations

	☐ 7 do not believe the influenza vaccine is safe<br>☐ 8 It is not recommended by my Ministry of Health or my organization<br>☐ 9 prefer natural immunity<br>☐ 10 Other (please explain)<br>……………………………………………..

III. Recommendation of COVID-19 Vaccines by Health Care Workers

III. Recommendation of COVID-19 Vaccines by Health Care Workers	Answer option
**C14. Did you recommend COVID-19 vaccines to your patients during the COVID-19 pandemic?**	☐ 1 Yes (skip to C16)<br>☐ 2 No
**C14.1 If no, why not?**<br>……………………………………………………………………………………………<br>……………………………………………………………………………………………<br>……………………………………………………………………………………………	
**C15. Do you now recommend COVID-19 vaccines and boosters to your patients?**	☐ 1 yes (skip to 16)<br>☐ 2 No
**C15.1 If no, why not?**<br>……………………………………………………………………………………………<br>……………………………………………………………………………………………<br>……………………………………………………………………………………………<br>………………………………………………	
**C16. If COVID-19 becomes an annually recommended vaccine, will you recommend annual vaccination to your patients?**	☐ 1 Yes<br>☐ 2 No (skip to C19)<br>☐ 3 I don't know
**C17. If yes, who are the groups of people you would vaccinate or would recommend they receive the vaccine against COVID-19? [INTERVIEWER: DO NOT READ LIST ALOUD]**	☐ 1 Healthy adults<br>☐ 2 Older adults (65 and older)<br>☐ 3 School age children (5-16 years old)<br>☐ 4 Children younger than 5<br>☐ 5 Women of childbearing age<br>☐ 6 Pregnant women<br>☐ 7 Health care workers<br>☐ 8 People with HIV/AIDS<br>☐ 9 Immunocompromised (from medication/treatments)<br>☐ 10 People with Tuberculosis<br>☐ 11 Persons with chronic medical conditions<br>☐ 12 I don't know

**C18. What is the most important (top) reason that would make you RECOMMEND the COVID-19 vaccine? (skip to 20)**	☐ 1. To protect my patients<br>☐ 2. To protect my family<br>☐ 3. It is easy/offered at my workplace<br>☐ 4. It is free<br>☐ 5. It is recommended by my Ministry of Health or my organization<br>☐ 6. Other reason (please explain)<br>……………………………………………<br>……………………………………………
**C19. What is the most important (top) reason that would make you NOT RECOMMEND the COVID-19 vaccine?**	……………………………………………<br>……………………………………………<br>……………………………………………<br>……………………………………………
**C20. Have you encountered any challenges when recommending COVID-19 vaccine to patients?**	☐ 1 Yes<br>☐ 2 No<br>☐ 3 I don't know
**C20.1 If yes, what reason(s) do you hear for the resistance or hesitancy to receive the COVID-19 vaccine? (select all that apply)**	☐ 1 Access<br>☐ 1) Availability<br>☐ 2) Location of clinic<br>☐ 3) Transportation<br>☐ 2 Time<br>☐ 1) Travel time to clinic<br>☐ 2) Waiting time<br>☐ 3 Vaccine cost<br>☐ 4 Visit cost/other cost<br>☐ 5 Patient fear of safety<br>☐ 6 Other (please explain)<br>..<br>..
**C21. Can the COVID-19 vaccine protect you against influenza infection?**	☐ 1 Yes<br>☐ 2 No<br>☐ 3 I don't know

SECTION 4: Experience with Influenza Vaccines

INTERVIEWER: Thank you for sharing your opinions on COVID-19. I will now ask some questions specifically related to your experience with influenza vaccination.

Experience	Answer option
**4.1. Did you receive the influenza vaccine *in the last season*?**	☐ 1 Yes<br>☐ 2 No (skip to D3)
**D1. If Yes, you received an influenza vaccine, did that influence your decision to accept the COVID-19 vaccine(s)?**	☐ 1 Yes (D2.1) please explain<br>………………………………………………<br>……………………………………………....<br>☐ 2 No (D2.2) please explain<br>………………………………………………<br>………………………………………………<br>☐ 3 I don't know (Skip to D6)
**D3. Did your experience with influenza vaccines influence your decision to recommend COVID-19 vaccines to your patients?**	☐ 1Yes (D4.1) Please explain why<br>..<br>..<br>☐ 2. No (D4.2) Please explain why not<br>..<br>..<br>☐ 3 I don't know
**D6. If the COVID-19 vaccine and seasonal influenza vaccine are offered together this influenza season (co-administered), will you accept both?**	☐ 1 Yes, I would receive both (Skip to E1)<br>☐ 2 No, I would not receive either<br>☐ 3 No, I would accept influenza vaccine ONLY<br>☐ 4 No, I would accept COVID-19 vaccine ONLY<br>☐ 5 I don't know<br>D6.1 Please explain why not<br>………………………………………………<br>………………………………………………

SECTION 5: Comments

Comments	Answer
**1. Do you have comments or suggestions regarding influenza vaccine for health care workers?**	……………………………………………………………<br>……………………………………………………………<br>……………………………………………………………
**2. Do you have comments or suggestions regarding COVID-19 vaccine for health care workers?**	……………………………………………………………<br>……………………………………………………………<br>…………………………………………………………..
