# Supplementary material for: Perceptions of influenza and SARS-CoV-2 vaccination among health care personnel in Thailand, 2024
Source: PLoS One. 2025 Aug 14;20(8):e0329473. doi: 10.1371/journal.pone.0329473 (PMC12352830; doi:10.1371/journal.pone.0329473)
Supplement: S2 File — Translated version used for interviews. (PDF) [file pone.0329473.s002.pdf]

ภาคผนวก 7 แบบสัมภาษณ์ที่ใช้ในการวิจัย

หมายเลข ☐☐☐☐☐☐

แบบสัมภาษณ์ การสำรวจ ความรู้ ทัศนคติ การปฏิบัติ และการรับรู้ ของบุคลากรทางการแพทย์

วันที่สัมภาษณ์..

ชื่อผู้สัมภาษณ์..

1. ชื่อ-สกุล ....บทบาท (ผู้สัมภาษณ์/ผู้บันทึกข้อมูล)
2. ชื่อ-สกุล ....บทบาท (ผู้สัมภาษณ์/ผู้บันทึกข้อมูล)
3. ชื่อ-สกุล ....บทบาท (ผู้สัมภาษณ์/ผู้บันทึกข้อมูล)
4. อื่น ๆ

(กรณีอื่น ๆ) โปรดระบุ ชื่อ-สกุล ผู้สัมภาษณ์..

สถานที่ตั้ง

☐ ประเทศไทย

ภูมิภาค

☐ 1 ภาคเหนือ

☐ 2 ภาคกลาง

☐ 3 ภาคใต้

☐ 4 ภาคตะวันออกเฉียงเหนือ

จังหวัดและอำเภอ

☐ 1 เชียงราย	☐ 1 อำเภอเมืองเชียงราย	☐ 1 อำเภอแม่สาย
☐ 2 พะเยา	☐ 2 อำเภอเมืองพะเยา	☐ 2 อำเภอดอกคำใต้
☐ 3 อุทัยธานี	☐ 3 อำเภอเมือง	☐ 3 อำเภอทัพทัน
☐ 4 ราชบุรี	☐ 4 อำเภอเมืองราชบุรี	☐ 4 อำเภอโพธาราม
☐ 5 นครพนม	☐ 5 อำเภอเมืองนครพนม	☐ 5 อำเภอโพนสวรรค์
☐ 6 หนองคาย	☐ 6 อำเภอเมืองหนองคาย	☐ 6 อำเภอท่าบ่อ
☐ 7 สุราษฎร์ธานี	☐ 7 อำเภอเมือง	☐ 7 อำเภอท่าฉาง

COE No.COE66/015
The Kasetsart University
Research Ethics Committee
Date of exemption 28 November 2023

☐ 8 ชุมพร ☐ 8 อำเภอเมืองชุมพร ☐ 8 อำเภอหลังสวน

ชนบท หรือในเมือง

☐ 1 ชนบท

☐ 2 ในเมือง

ประเภทของสถานบริการ

☐ 1 โรงพยาบาลจังหวัด

☐ 2 โรงพยาบาลชุมชน

ชื่อของสถานบริการ

☐ 1 โรงพยาบาลเชียงรายประชานุเคราะห์

☐ 2 โรงพยาบาลพะเยา

☐ 3 โรงพยาบาลอุทัยธานี

☐ 4 โรงพยาบาลราชบุรี

☐ 5 โรงพยาบาลนครพนม

☐ 6 โรงพยาบาลหนองคาย

☐ 7 โรงพยาบาลสุราษฎร์ธานี

☐ 8 โรงพยาบาลชุมพรเขตรอุดมศักดิ์

☐ 9 โรงพยาบาลแม่สาย

☐ 10 โรงพยาบาลดอกคำใต้

☐ 11 โรงพยาบาลทัพทัน

☐ 12 โรงพยาบาลโพธาราม

☐ 13 โรงพยาบาลโพนสวรรค์

☐ 14 โรงพยาบาลสมเด็จพระยุพราชท่าบ่อ

☐ 15 โรงพยาบาลท่าฉาง

☐ 16 โรงพยาบาลหลังสวน

ใน (ประเทศไทย) มีนโยบายการฉีดวัคซีนไข้หวัดใหญ่ หรือไม่

☐ 1 มี เฉพาะในบุคลากรทางการแพทย์เท่านั้น

☐ 2 มี สำหรับประชากรกลุ่มเสี่ยง (ข้อแนะนำของประเทศ)

☐ 3 ไม่มี

☐ 4 ไม่ทราบ/ไม่แน่ใจ

ท่านสามารถรับวัคซีนไข้หวัดใหญ่ได้ หรือไม่ หากต้องการ

☐ 1 ได้

☐ 2 ไม่ได้

☐ 3 ไม่ทราบ/ไม่แน่ใจ

ส่วนที่ 1: คำถามทั่วไป และข้อมูลประชากร

COE No.COE66/015
The Kasetsart University
Research Ethics Committee
KASETSART UNIVERSITY 1943
Date of exemption 28 November 2023

ผู้สัมภาษณ์: ขอขอบคุณท่านที่สละเวลา ฉันขอเริ่มต้นการสอบถามข้อมูลทั่วไป และข้อมูลประชากร

คำแนะนำ: โปรดตอบคำถามโดยทำเครื่องหมาย ✓ในช่อง ☐ ที่เลือก

คำถามทั่วไป และข้อมูลประชากร	ตัวเลือกคำตอบ
1. เพศ	☐ 1 ชาย<br>☐ 2 หญิง<br>☐ 3 ไม่ประสงค์ตอบ
2. อายุ (ปี)	☐ 1 18 – 24 ปี<br>☐ 2 25 – 29 ปี<br>☐ 3 30 – 34 ปี<br>☐ 4 35 – 39 ปี<br>☐ 5 40 – 49 ปี<br>☐ 6 50 – 59 ปี<br>☐ 7 60 ปี ขึ้นไป
3. ท่านทำงานในโรงพยาบาล มาแล้วกี่ปี	☐ 1 น้อยกว่า 1 ปี<br>☐ 2 1 - 4 ปี<br>☐ 3 5 - 9 ปี<br>☐ 4 10 - 14 ปี<br>☐ 5 15 - 19 ปี<br>☐ 6 20 - 24 ปี<br>☐ 7 25 - 29 ปี<br>☐ 8 30 ปี ขึ้นไป
4. อาชีพของท่าน คืออะไร	☐ 1 แพทย์<br>☐ 2 ผู้ช่วยแพทย์<br>☐ 3 พยาบาล<br>☐ 4 ผู้ช่วยพยาบาล<br>☐ 5 ผดุงครรภ์

COE No.COE66/015
The Kasetsart University
Research Ethics Committee
KASETSART UNIVERSITY 1943
Date of exemption 28 November 2023

	☐ 6 ทันตแพทย์<br>☐ 7 เภสัชกร<br>☐ 8 เจ้าหน้าที่สาธารณสุข<br>☐ 9 เจ้าหน้าที่การแพทย์ฉุกเฉิน (EMT)/กู้ชีพ<br>☐ 10 เจ้าหน้าที่ห้องปฏิบัติการ<br>☐ 11 ผู้ช่วยเหลือคนไข้<br>☐ 12 อื่น ๆ (โปรดระบุ)......................................
5. ท่านทำงานที่แผนกใด ในโรงพยาบาล	☐ 1 ทั่วไป/ อายุรกรรม<br>☐ 2 ผู้ป่วยหนักผู้ใหญ่<br>☐ 3 ผู้ป่วยหนักทารกแรกเกิด<br>☐ 4 แผนกฉุกเฉิน<br>☐ 5 สูติ-นรีเวช<br>☐ 6 เด็ก<br>☐ 7 รังสีวิทยา<br>☐ 8 ศัลยกรรม<br>☐ 9 อื่น ๆ (โปรดระบุ)..
6. โดยทั่วไปผู้ป่วยประเภทใด ที่ท่านให้การดูแล	☐ 1 หญิงตั้งครรภ์<br>☐ 2 เด็ก<br>☐ 3 ผู้ใหญ่ที่มีโรคเรื้อรัง<br>☐ 4 ผู้ใหญ่โรคติดเชื้อ<br>☐ 5 ผู้สูงอายุ (มากกว่า 65 ปี)<br>☐ 6 อื่น ๆ (โปรดระบุ)..
7 ก. มีแพทย์หรือเจ้าหน้าที่สาธารณสุขคนอื่นบอกท่านหรือไม่ว่า ท่านเป็นผู้มีภาวะสุขภาพโรคเรื้อรังในระยะยาว ซึ่งอาจรวมถึง โรคอ้วน เบาหวาน โรคปอด ความดันโลหิตสูง หรือภาวะอื่น ๆ ในระยะยาว	☐ 1 ใช่<br>☐ 2 ไม่ใช่
7 ข. ถ้าใช่ ภาวะสุขภาพเรื้อรังในระยะยาว คืออะไร	☐ 1 โรคอ้วน

COE No.COE66/015
The Kasetsart University
Research Ethics Committee
KASETSART UNIVERSITY 1943
Date of exemption ..28 November 2023..........

(อย่าอ่านตัวเลือกตอบ เลือกตอบได้มากกว่าหนึ่งข้อ)	☐ 2 โรคเบาหวาน<br>☐ 3 โรคหัวใจ<br>☐ 4 โรคปอด<br>☐ 5 โรคภูมิคุ้มกันบกพร่อง<br>☐ 6 โรคเรื้อรังอื่น ๆ (โปรดระบุ).........................

ส่วนที่ 2 โรคไข้หวัดใหญ่ และวัคซีนป้องกันไข้หวัดใหญ่

ผู้สัมภาษณ์: ขอขอบคุณสำหรับการแบ่งปันข้อมูลประชากรของท่าน ตอนนี้ ฉันจะถามคำถามที่เกี่ยวข้องกับโรคไข้หวัดใหญ่และการฉีดวัคซีน

I. ความรู้ทั่วไป และการรับรู้

คำแนะนำ: โปรดตอบคำถามโดยทำเครื่องหมาย ✓ ในช่อง ☐ ที่เลือก

**I. ความรู้ทั่วไป และการรับรู้**	**ตัวเลือกคำตอบ**
1. ท่านเคยวินิจฉัยผู้ป่วยเป็นโรคไข้หวัดใหญ่ โดยใช้อาการหรือใช้การตรวจทางห้องปฏิบัติการ หรือไม่	☐ 1 เคย<br>☐ 2 ไม่เคย<br>☐ 3 จำไม่ได้
2. ท่านเคยให้การดูแลรักษาผู้ป่วยที่มีอาการรุนแรง เช่น ปอดอักเสบหรือเสียชีวิต ที่เป็นผู้นอนพักรักษาตัวในโรงพยาบาล เนื่องจากป่วยเป็นโรคไข้หวัดใหญ่ หรือสงสัยว่าป่วยเป็นโรคไข้หวัดใหญ่ หรือไม่	☐ 1 เคย<br>☐ 2 ไม่เคย<br>☐ 3 จำไม่ได้

รายละเอียดด้านล่าง คือ ข้อคำถามเกี่ยวกับความรุนแรงของโรค โอกาสเสี่ยงของการเป็นโรค และความรุนแรงของโรคไข้หวัดใหญ่ ความปลอดภัย และประสิทธิผลของวัคซีนป้องกันไข้หวัดใหญ่ ในประชากรกลุ่มต่าง ๆ โปรดทำเครื่องหมาย ✓ ในช่อง ที่ท่านเห็นด้วยหรือไม่เห็นด้วยมากน้อยเพียงใดกับข้อความในแต่ละข้อ

3. ความรุนแรงของโรคไข้หวัดใหญ่: ในทุกปี โรคไข้หวัดใหญ่เป็นสาเหตุทำให้กลุ่มคนเหล่านี้ ต้องนอนพักรักษาตัว ในโรงพยาบาล อยู่ในหอผู้ป่วยหนัก (ICU) หรือเสียชีวิตได้ สำหรับ (พูดถึงกลุ่มคนที่เฉพาะเจาะจงด้านล่าง) ท่านเห็นด้วยอย่างยิ่ง เห็นด้วย ไม่เห็นด้วย หรือไม่เห็นด้วยอย่างยิ่ง

**กลุ่มประชากร**	**เห็นด้วยอย่างยิ่ง**	**เห็นด้วย**	**ไม่เห็นด้วย**	**ไม่เห็นด้วยอย่างยิ่ง**	**ไม่มีความคิดเห็น**

COE No. COE66/015
The Kasetsart University
Research Ethics Committee
1943
Date of exemption 28 November 2023

บุคลากรทางการแพทย์					
ประชาชนทั่วไป					
หญิงตั้งครรภ์					
ทารกในครรภ์					
ผู้ที่มีโรคประจำตัวเรื้อรัง					
ผู้ที่มีอายุตั้งแต่ 65 ปี ขึ้นไป					
เด็กอายุน้อยกว่า 5 ปี					
เด็กวัยเรียน					
ผู้ตอบแบบสัมภาษณ์					

4. โอกาสเสี่ยงและความรุนแรง: เมื่อติดเชื้อโรคไข้หวัดใหญ่จนทำให้ต้องนอนพักรักษาตัวในโรงพยาบาล อยู่ในหอผู้ป่วยหนักหรือเสียชีวิต ในแต่ละปี มีความเป็นไปได้มากแค่ไหนที่กลุ่มคนเหล่านี้ (พูดถึงกลุ่มคนเฉพาะเจาะจงด้านล่าง) มีประสบการณ์การติดเชื้อไข้หวัดใหญ่จนต้องพบแพทย์ มีความเป็นไปได้มาก เป็นไปได้ ไม่น่าเป็นไปได้ หรือไม่น่าเป็นไปได้มาก

กลุ่มประชากร	เป็นไปได้มาก	เป็นไปได้	ไม่น่าเป็นไปได้	ไม่น่าเป็นไปได้มาก	ไม่มีความคิดเห็น
บุคลากรทางการแพทย์					
ประชาชนทั่วไป					
หญิงตั้งครรภ์					
ผู้ที่มีโรคประจำตัวเรื้อรัง					
ผู้ที่มีอายุตั้งแต่ 65 ปี ขึ้นไป					
เด็กอายุน้อยกว่า 5 ปี					
เด็กวัยเรียน					
ตัวผู้ตอบแบบสัมภาษณ์เอง					

5. ความปลอดภัยของวัคซีนป้องกันไข้หวัดใหญ่: ท่านเชื่อว่าวัคซีนป้องกันโรคไข้หวัดใหญ่มีความปลอดภัยมากน้อยเพียงใด สำหรับกลุ่มคนเหล่านี้ (พูดถึงกลุ่มคนเฉพาะเจาะจงด้านล่าง) ท่านเชื่อว่าปลอดภัยมา ปลอดภัย ไม่ปลอดภัย หรือไม่ปลอดภัยมาก

กลุ่มประชากร	เห็นด้วยอย่างยิ่ง	เห็นด้วย	ไม่เห็นด้วย	ไม่เห็นด้วยอย่างยิ่ง	ไม่มีความคิดเห็น
บุคลากรทางการแพทย์					

COE No.COE66/015
The Kasetsart University
Research Ethics Committee
KASETSART UNIVERSITY 1943
Date of exemption 28 November 2023

ประชาชนทั่วไป					
หญิงตั้งครรภ์					
ทารกในครรภ์					
ผู้ที่มีโรคประจำตัวเรื้อรัง					
ผู้ที่มีอายุตั้งแต่ 65 ปี ขึ้นไป					
เด็กอายุน้อยกว่า 5 ปี					
เด็กวัยเรียน					
ตัวผู้ตอบแบบสัมภาษณ์เอง					

6. ประสิทธิผลของวัคซีนป้องกันไข้หวัดใหญ่: การได้รับวัคซีนป้องกันไข้หวัดใหญ่สามารถลดโอกาสที่จะเกิดความรุนแรงจากโรคไข้หวัดใหญ่ เช่น ต้องเข้านอนพักรักษาตัวในโรงพยาบาล ในกลุ่มคนเหล่านี้ (พูดถึงกลุ่มคนที่เฉพาะเจาะจงด้านล่าง) ท่านเห็นด้วยอย่างยิ่ง เห็นด้วย ไม่เห็นด้วย หรือไม่เห็นด้วยอย่างยิ่ง

**กลุ่มประชากร**	**ปลอดภัยมาก**	**ปลอดภัย**	**ไม่ปลอดภัย**	**ไม่ปลอดภัยมาก**	**ไม่มีความคิดเห็น**
บุคลากรทางการแพทย์					
ประชาชนทั่วไป					
หญิงตั้งครรภ์					
ทารกในครรภ์					
ผู้ที่มีโรคประจำตัวเรื้อรัง					
ผู้ที่มีอายุตั้งแต่ 65 ปี ขึ้นไป					
เด็กอายุน้อยกว่า 5 ปี					
เด็กวัยเรียน					
ตัวผู้ตอบแบบสัมภาษณ์เอง					

7. วัคซีนป้องกันโรคไข้หวัดใหญ่สามารถป้องกัน ไม่ให้ท่านเจ็บป่วยหรือติดเชื้อโรคโควิด-19 ได้

☐ 1 เชื่อ

☐ 2 ไม่เชื่อ

☐ 3 ไม่ทราบ/ไม่แน่ใจ

**II. 2 การยอมรับวัคซีนป้องกันไข้หวัดใหญ่ของบุคลากรทางการแพทย์**	ตัวเลือกคำตอบ

COE No.COE66/015
The Kasetsart University
Research Ethics Committee
KASETSART UNIVERSITY 1943
Date of exemption 28 November 2023

8. ท่านจำการระบาดใหญ่ครั้งล่าสุดได้ หรือไม่ ไข้หวัดใหญ่สายพันธุ์ เอช วัน เอ็น วัน เกิดการระบาด ในปี ค.ศ. 2009	☐ 1 ใช่<br>☐ 2 ไม่ใช่<br>☐ 3 ไม่ทราบ
9. ถ้าใช่ ท่านได้รับการฉีดวัคซีนในช่วงการระบาดของไข้หวัดใหญ่ 2009 หรือไม่	☐ 1 ใช่<br>☐ 2 ไม่ใช่<br>☐ 3 จำไม่ได้/ ไม่แน่ใจ
10. ท่านเคยได้รับวัคซีนไข้หวัดใหญ่ ก่อนที่จะเริ่มมีการระบาดของโรคโควิด-19 หรือไม่	☐ 1 ใช่<br>☐ 2 ไม่ใช่<br>☐ 3 จำไม่ได้/ ไม่แน่ใจ (ข้ามไป คำถามข้อ 12)
11. ถ้าใช่ หรือไม่ใช่ โปรดอธิบายว่าทำไมใช่ หรือทำไมไม่ใช่<br>..<br>..	
12. ฤดูกาลไข้หวัดใหญ่ที่ผ่านมา ท่านได้รับวัคซีนไข้หวัดใหญ่ หรือไม่	☐ 1 ได้รับ<br>☐ 2 ไม่ได้รับ (ข้ามไป คำถามข้อ 12.2)<br>☐ 3 จำไม่ได้/ ไม่แน่ใจ (ข้ามไป คำถามข้อ 13)
12.1 ถ้าเคยได้รับวัคซีน อะไรที่ทำให้ท่านตัดสินใจรับวัคซีน คือ (เลือกได้มากกว่า 1 ข้อ)	☐ 1 รับวัคซีนในทุกปี<br>☐ 2 ได้รับวัคซีนด้วยความสมัครใจ ในโรงพยาบาลโดยไม่เสียค่าใช้จ่าย<br>☐ 3 เป็นข้อบังคับของโรงพยาบาล ให้เจ้าหน้าที่ฉีดวัคซีนป้องกันไข้หวัดใหญ่<br>☐ 4 เพื่อป้องกันไม่ให้ผู้ป่วยติดเชื้อโรคไข้หวัดใหญ่<br>☐ 5 เพื่อป้องกันตัวเองไม่ให้ติดเชื้อโรคไข้หวัดใหญ่<br>☐ 6 เพื่อป้องกันครอบครัว<br>☐ 7 เข้าถึงวัคซีนได้ง่าย/ มีการฉีดให้ในโรงพยาบาล<br>☐ 8 ไม่เสียค่าใช้จ่าย

KASETSART UNIVERSITY 1943
COE No.COE66/015
The Kasetsart University
Research Ethics Committee
Date of exemption 28 November 2023

	☐ 9 กระทรวงสาธารณสุขหรือโรงพยาบาลของฉันแนะนำให้ฉีดวัคซีน<br>☐ 10 เหตุผลอื่น ๆ (โปรดระบุ)<br>………………………………………………………………………………<br>………………………………………………………………………………
12.2 ถ้าไม่เคยได้รับวัคซีน ท่านมีเหตุอะไรที่ทำให้ท่านตัดสินใจไม่ฉีดวัคซีน (เลือกได้มากกว่า 1 ข้อ)	☐ 1 ในประเทศไทย ไม่มีการให้บริการให้วัคซีนป้องกันโรคไข้หวัดใหญ่แก่บุคลากรการแพทย์<br>☐ 2 ฉันไม่เชื่อว่าวัคซีนป้องกันไข้หวัดใหญ่สามารถป้องกันการติดเชื้อได้<br>☐ 3 ไม่ต้องการเสียค่าใช้จ่ายในการฉีดวัคซีน (ในโรงพยาบาลฉีดวัคซีนไข้หวัดใหญ่ต้องเสียค่าใช้จ่าย)<br>☐ 4 ไม่มีเวลาไปฉีดวัคซีน<br>☐ 5 ฉันไม่เชื่อว่าตนเองเป็นกลุ่มเสี่ยงต่อการติดเชื้อโรคไข้หวัดใหญ่<br>☐ 6 ฉันไม่เชื่อในวัคซีนป้องกันไข้หวัดใหญ่<br>☐ 7 ไม่เชื่อว่าวัคซีนไข้หวัดใหญ่มีความปลอดภัย<br>☐ 8 กระทรวงสาธารณสุขหรือโรงพยาบาลของฉันไม่ได้แนะนำให้ฉีดวัคซีนป้องกันไข้หวัดใหญ่<br>☐ 9 ให้ความสำคัญกับภูมิคุ้มกันที่มีอยู่ตามธรรมชาติมากกว่า<br>☐ 10 ฉีดวัคซีนมีความไม่สะดวก มีความยุ่งยากในการรับบริการ<br>☐ 11 วัคซีนมีจำนวนน้อย ไม่เพียงพอ<br>☐ 12 วัคซีนมีผลข้างเคียงหรืออาการไม่พึงประสงค์<br>☐ 13 วัคซีนทำให้ฉันป่วย<br>☐ 14 ไม่ชอบการฉีดวัคซีน กลัวเจ็บ

COE No.COE66/015
The Kasetsart University
Research Ethics Committee
KASETSART UNIVERSITY 1943
Date of exemption 28 November 2023

	☐ 15 อื่น ๆ (โปรดอธิบายเพิ่มเติม)<br>...<br>...
13. หากวัคซีนป้องกันไข้หวัดใหญ่ แนะนำให้ฉีดในกลุ่มบุคลากรทางการแพทย์ โดยไม่เสียค่าใช้จ่าย ท่านจะฉีดวัคซีน หรือไม่	☐ 1 ฉีด<br>☐ 2 ไม่ฉีด (ข้ามไปตอบ ข้อ 15)<br>☐ 3 ไม่ทราบ
14. เหตุผลที่สำคัญที่สุด ที่ทำให้ท่านตัดสินใจฉีดวัคซีนป้องกันไข้หวัดใหญ่ คืออะไร (ระบุแล้ว ให้ข้ามไปตอบข้อ 16)	....<br>...<br>...
15. เหตุผลที่สำคัญที่สุด ที่ทำให้ท่านตัดสินใจไม่ฉีดวัคซีนป้องกันไข้หวัดใหญ่ คืออะไร	....<br>...<br>...

III. 3 การให้คำแนะนำวัคซีนป้องกันไข้หวัดใหญ่ โดยบุคลากรทางการแพทย์

**III. 3 การให้คำแนะนำวัคซีนป้องกันไข้หวัดใหญ่ โดยบุคลากรทางการแพทย์**	**ตัวเลือกคำตอบ**
16. หากวัคซีนป้องกันไข้หวัดใหญ่ มีบริการสำหรับผู้ป่วย ท่านจะฉีดวัคซีนให้ หรือให้คำแนะนำผู้ป่วยฉีดวัคซีนป้องกันไข้หวัดใหญ่ หรือไม่	☐ 1 ใช่<br>☐ 2 ไม่ใช่ (ข้ามไปตอบ ข้อ 19)
17. ถ้าท่านแนะนำให้ฉีดวัคซีน<br>กลุ่มคนกลุ่มใดที่ท่านจะฉีดวัคซีนหรือให้คำแนะนำกลุ่มคนเหล่านั้นฉีดวัคซีนป้องกันไข้หวัดใหญ่<br>(ผู้สัมภาษณ์: ไม่อ่านออกเสียง)	☐ 1 ผู้ใหญ่ที่มีสุขภาพแข็งแรง<br>☐ 2 ผู้ที่มีอายุตั้งแต่ 65 ปี ขึ้นไป<br>☐ 3 เด็กวัยเรียน<br>☐ 4 เด็กอายุน้อยกว่า 5 ปี<br>☐ 5 ผู้หญิงที่สามารถมีบุตรได้<br>☐ 6 หญิงตั้งครรภ์<br>☐ 7 บุคลากรการแพทย์

COE No.COE66/015
KASETSART UNIVERSITY 1943
The Kasetsart University
Research Ethics Committee
Date of exemption 28 November 2023

	☐ 8 ผู้ติดเชื้อ เอช ไอ วี/ เอดส์<br>☐ 9 ผู้ป่วยโรควัณโรค<br>☐ 10 ผู้ที่มีโรคประจำตัวเรื้อรัง<br>☐ 11 ไม่ทราบ
18. เหตุผลที่สำคัญที่สุด ที่ทำให้ท่าน**<u>ให้คำแนะนำ</u>**วัคซีนป้องกันโรคไข้หวัดใหญ่ (ระบุแล้ว ให้ข้ามไปตอบข้อ 20)	....<br>..<br>..
19. เหตุผลสำคัญ ที่ทำให้ท่าน**<u>ไม่ให้คำแนะ</u>**นำวัคซีนป้องกันโรคไข้หวัดใหญ่	....<br>..
20. ท่านเคยเผชิญกับกรณีที่ผู้ป่วยต่อต้านหรือลังเลที่จะรับวัคซีนป้องกันไข้หวัดใหญ่ หรือไม่	☐ 1 เคย<br>☐ 2 ไม่เคย (ข้ามไปตอบ ส่วนที่ 3)
20.1. <u>ถ้าเคย</u> ข้ามไป เหตุผลที่ต่อต้านหรือลังเลที่จะรับวัคซีนป้องกันไข้หวัดใหญ่ คืออะไร (ตอบได้มากกว่า 1 ข้อ)	☐ 1 เข้าถึงวัคซีน<br>☐ 1) ความพร้อมของวัคซีน<br>☐ 2) สถานที่ฉีด<br>☐ 3) การเดินทาง<br>☐ 2 เวลา<br>☐ 1) เวลาเดินทางไปคลินิก<br>☐ 2) ระยะเวลารอ (เช่น ต่อคิวฉีดวัคซีน)<br>☐ 3 ค่าวัคซีน<br>☐ 4 ค่าบริการ/ค่าใช้จ่ายอื่นๆ<br>☐ 5 ผู้ป่วยกลัวเรื่องความปลอดภัย<br>☐ 6 อื่นๆ (โปรดอธิบาย..<br>...

ส่วนที่ 3: โรคโควิด – 19 และวัคซีนป้องกันโรคโควิด - 19

ผู้สัมภาษณ์: ขอขอบคุณสำหรับการแบ่งปันความคิดเห็นของท่านเกี่ยวกับโรคไข้หวัดใหญ่และการฉีดวัคซีน ตอนนี้ฉันจะถามบางคำถามที่เกี่ยวข้องกับโรคโควิด-19 และการฉีดวัคซีน

COE No.COE66/015
The Kasetsart University
Research Ethics Committee
Date of exemption 28 November 2023

I. ความรู้ทั่วไป และทัศนคติ

**I. ความรู้ทั่วไป และทัศนคติ**	**ตัวเลือกคำตอบ**
1. ท่านเคยวินิจฉัยผู้ป่วยเป็นโรคโควิด-19 จากอาการทางคลินิกหรือโดยใช้ผลตรวจยืนยันจากห้องปฏิบัติการหรือไม่	☐ 1 เคย<br>☐ 2 ไม่เคย<br>☐ 3 จำไม่ได้
2. ท่านเคยให้การรักษาผู้ป่วยโรคโควิด– 19 ที่มีภาวะแทรกซ้อนที่คุกคามถึงชีวิต (เช่น โรคปอดอักเสบหรือเสียชีวิต) และต้องเข้าพักรักษาตัวในโรงพยาบาลเนื่องจากป่วยเป็นโรคโควิด–19 หรือสงสัยว่าป่วยเป็นโรคโควิด–19 หรือไม่	☐ 1 เคย<br>☐ 2 ไม่เคย<br>☐ 3 จำไม่ได้

รายละเอียดด้านล่าง คือ ชุดข้อมูลเกี่ยวกับความรุนแรงของโรคโควิด-19 ความปลอดภัย และประสิทธิผลของวัคซีนป้องกันโรคโควิด-19

โปรดทำเครื่องหมาย ✓ ในช่องที่ท่านเห็นด้วยหรือไม่เห็นด้วย มากน้อยเพียงใด ในแต่ละข้อ

3. ความรุนแรงของโรคโควิด-19: โรคโควิด-19 เป็นสาเหตุทำให้เกิดการเจ็บป่วยรุนแรงจนต้องเข้านอนพักรักษาตัวในโรงพยาบาล ต้องให้การดูแลระยะยาวหรือเสียชีวิตในกลุ่มสมาชิกที่ไม่ได้รับการฉีดวัคซีน ดังต่อไปนี้ ท่านเห็นด้วยอย่างยิ่ง เห็นด้วย ไม่เห็นด้วย หรือไม่เห็นด้วยอย่างยิ่ง

**กลุ่มประชากร**	**เห็นด้วยอย่างยิ่ง**	**เห็นด้วย**	**ไม่เห็นด้วย**	**ไม่เห็นด้วยอย่างยิ่ง**	**ไม่แน่ใจ**
บุคลากรทางการแพทย์					
ประชาชนทั่วไป					
หญิงตั้งครรภ์					
ผู้ที่มีโรคประจำตัวเรื้อรัง					
ผู้ที่มีอายุตั้งแต่ 65 ปี ขึ้นไป					
ผู้ที่มีภาวะภูมิคุ้มกันบกพร่อง					
กลุ่มเปราะบาง ด้อยโอกาส					
เด็กและวัยรุ่น					
ตัวผู้ตอบแบบสัมภาษณ์เอง					

COE No.COE66/015
The Kasetsart University
Research Ethics Committee
KASETSART UNIVERSITY 1943
Date of exemption 28 November 2023

4. ความปลอดภัยของวัคซีน: ท่านเชื่อว่าวัคซีนป้องกันโรค โควิด-19 มีความปลอดภัยแค่ไหนมากน้อยแค่ไหนสำหรับกลุ่มคน ดังต่อไปนี้ ปลอดภัยมาก ปลอดภัย ไม่ปลอดภัยหรือไม่ปลอดภัยมาก

**กลุ่มประชากร**	**ปลอดภัยมาก**	**ปลอดภัย**	**ไม่ปลอดภัย**	**ไม่ปลอดภัยมาก**	**ไม่แน่ใจ**
บุคลากรทางการแพทย์					
ประชาชนทั่วไป					
หญิงตั้งครรภ์					
ผู้ที่มีโรคประจำตัวเรื้อรัง					
ผู้ที่มีอายุตั้งแต่ 65 ปี ขึ้นไป					
ผู้ที่มีภาวะภูมิคุ้มกันบกพร่อง					
กลุ่มเปราะบาง ด้อยโอกาส					
เด็กและวัยรุ่น					
ตัวผู้ตอบแบบสัมภาษณ์เอง					

5. ประสิทธิผลของวัคซีนโควิด-19: การได้รับวัคซีนป้องกันโรคโควิด-19 สามารถลดโอกาสที่จะเจ็บป่วยรุนแรงด้วยโรคโควิด-19 ได้มาก (เช่น ต้องเข้าพักรักษาตัวในโรงพยาบาล) สำหรับกลุ่มคน ดังต่อไปนี้ ท่านเห็นด้วยอย่างยิ่ง เห็นด้วย ไม่เห็นด้วย หรือไม่เห็นด้วยอย่างยิ่ง

**กลุ่มประชากร**	**เห็นด้วยอย่างยิ่ง**	**เห็นด้วย**	**ไม่เห็นด้วย**	**ไม่เห็นด้วยอย่างยิ่ง**	**ไม่แน่ใจ**
บุคลากรทางการแพทย์					
ประชาชนทั่วไป					
หญิงตั้งครรภ์					
ผู้ที่มีโรคประจำตัวเรื้อรัง					
ผู้ที่มีอายุตั้งแต่ 65 ปี ขึ้นไป					
ผู้ที่มีภาวะภูมิคุ้มกันบกพร่อง					
กลุ่มเปราะบาง ด้อยโอกาส					
เด็กและวัยรุ่น					
ผู้ตอบแบบสัมภาษณ์					

COE No.COE66/015
The Kasetsart University
Research Ethics Committee
KASETSART UNIVERSITY 1943
Date of exemption 28 November 2023

II. การยอมรับวัคซีนป้องกันโรคโควิด -19 ของบุคลากรทางการแพทย์

**II. การยอมรับวัคซีนป้องกันโรคโควิด -19 ของบุคลากรทางการแพทย์**	**ตัวเลือกคำตอบ**
6. ท่านเคยได้รับการฉีดวัคซีนป้องกันโรคโควิด-19 ตั้งแต่เริ่มต้นให้มีการฉีดวัคซีนในประเทศ หรือไม่	☐ 1 เคย<br>☐ 2 ไม่เคย<br>☐ 3 จำไม่ได้
7. หากวัคซีนป้องกันโรคโควิด-19 กำหนดให้ฉีดมากกว่า 1 เข็ม ท่านได้รับวัคซีนครบตามจำนวนเข็มที่กำหนดไว้ หรือไม่	☐ 1 ได้รับครบ (ข้ามไปตอบ ข้อ 9)<br>☐ 2 ได้รับไม่ครบ<br>☐ 3 ไม่ทราบ
8. หากท่านได้รับการฉีดวัคซีนป้องกันโรคโควิด-19 เพียงครั้งเดียวจากจำนวนหลายเข็มที่กำหนดต้องฉีด โปรดอธิบายว่า ทำไมท่านจึงไม่ได้รับการฉีดเพิ่ม<br>..<br>..<br>..	
9. ท่านได้รับการฉีดวัคซีนป้องกันโรคโควิด-19 เข็มกระตุ้น อย่างน้อย 1 เข็ม หลังจากฉีดวัคซีนหลักครบแล้ว หรือไม่	☐ 1 ได้รับ<br>☐ 2 ไม่ได้รับ (ข้ามไปตอบ ข้อ 9.2)<br>☐ 3 ไม่ทราบ
9.1. ถ้าได้รับ ท่านได้รับวัคซีนฉีดเข็มกระตุ้นกี่เข็ม	☐ 1 จำนวน..................... เข็ม<br>☐ 2 จำไม่ได้
9.2. ถ้าไม่ได้รับ วัคซีนฉีดเข็มกระตุ้น เป็นเพราะเหตุใด<br>..<br>..<br>..	
10. ในปัจจุบัน องค์การอนามัยโลกแนะนำให้บุคลากรทางการแพทย์ด่านหน้า ฉีดวัคซีนป้องกันโรคโควิด-19 อย่างต่อเนื่อง หากในประเทศไทยยังดำเนินตามข้อแนะนำดังกล่าวนี้อยู่ ท่านจะฉีดวัคซีนทุกปี หรือไม่	☐ 1 ฉีด<br>☐ 2 ไม่ฉีด<br>☐ 3 ไม่ทราบ
11. หากวัคซีนป้องกันโรคโควิด-19 กลายเป็นวัคซีน	☐ 1 ฉีด

COE No.COE66/015
The Kasetsart University
Research Ethics Committee
Date of exemption ...28 November 2023...

ที่แนะนำให้บุคลากรทางการแพทย์ฉีดทุกปีเหมือนกับวัคซีนป้องกันไข้หวัดใหญ่ตามฤดูกาล ท่านจะรับการฉีดวัคซีนป้องกันโรคโควิด-19 ทุกปี หรือไม่	☐ 2 ไม่ฉีด<br>☐ 3 ไม่ทราบ
12. เหตุผลที่สำคัญที่สุด ที่ทำให้ท่าน**ยอมรับ**และฉีดวัคซีนป้องกันโรคโควิด-19 เข็มกระตุ้น หรือฉีดวัคซีนป้องกันโรคโควิด-19 เป็นประจำปี คืออะไร (ข้ามไปตอบ ข้อ 14)	☐ 1 ได้รับวัคซีนด้วยความสมัครใจ ในโรงพยาบาลโดยไม่เสียค่าจ่าย<br>☐ 2 เป็นข้อบังคับของโรงพยาบาล ให้เจ้าหน้าที่ฉีดวัคซีนป้องกันโรคโควิด-19<br>☐ 3 เพื่อปกป้องไม่ให้ผู้ป่วยติดเชื้อโรคโควิด-19<br>☐ 4 เพื่อป้องกันตนเองจากการติดเชื้อโรคโควิด-19<br>☐ 5 เพื่อปกป้องครอบครัว<br>☐ 6 เข้าถึงวัคซีนได้ง่าย/มีการให้ฉีดในโรงพยาบาล<br>☐ 7 ไม่เสียค่าใช้จ่าย<br>☐ 8 กระทรวงสาธารณสุขหรือโรงพยาบาลของฉันแนะนำให้ฉีด<br>☐ 9 อื่น (โปรดอธิบาย)..
13. เหตุผลที่สำคัญที่สุด ที่ทำให้ท่าน**ปฏิเสธการฉีด**วัคซีนป้องกันโรคโควิด-19 เข็มกระตุ้น หรือฉีดวัคซีนป้องกันโรคโควิด-19 เป็นประจำปี คืออะไร	☐ 1 ในประเทศไทย ไม่มีการให้บริการให้วัคซีนป้องกันโรคโควิด-19 แก่บุคลากรทางการแพทย์<br>☐ 2 ฉันไม่เชื่อว่าวัคซีนป้องกันโรคโควิด-19 สามารถป้องกันการติดเชื้อได้<br>☐ 3 ไม่ต้องการเสียค่าใช้จ่ายในการฉีดวัคซีน (ในโรงพยาบาลฉีดวัคซีนโรคโควิด-19 ต้องเสียค่าใช้จ่าย)<br>☐ 4 ไม่มีเวลาไปฉีดวัคซีน<br>☐ 5 ฉันไม่เชื่อว่าตัวเองเป็นกลุ่มเสี่ยงต่อการติดเชื้อโรคโควิด-19<br>☐ 6 ฉันไม่เชื่อในการฉีดวัคซีนโรคโควิด-19<br>☐ 7 ไม่เชื่อว่าวัคซีนโรคโควิด-19 มีความปลอดภัย

COE No.COE66/015
The Kasetsart University
Research Ethics Committee
Date of exemption 28 November 2023

	☐ 8 กระทรวงสาธารณสุขหรือโรงพยาบาลของฉันไม่ได้แนะนำให้ฉีดวัคซีนป้องกันโรคโควิด-19<br>☐ 9 ให้ความสำคัญกับภูมิคุ้มกันที่มีอยู่ตามธรรมชาติมากกว่า<br>☐ 10 อื่นๆ (โปรดอธิบาย) ....

III. การให้คำแนะนำวัคซีนป้องกันโรคโควิด-19 โดยบุคลากรทางการแพทย์

**III. 3 การให้คำแนะนำวัคซีนป้องกันโรคโควิด-19 โดยบุคลากรทางการแพทย์**	**ตัวเลือกคำตอบ**
14. ท่านได้ให้คำแนะนำวัคซีนป้องกันโรคโควิด-19 แก่ผู้ป่วยในช่วงที่มีการระบาดใหญ่ของโรคโควิด-19 หรือไม่	☐ 1 ให้ (ข้ามไปตอบ ข้อ 15)<br>☐ 2 ไม่ให้
14.1 ถ้าไม่ได้แนะนำ เป็นเพราะเหตุผลใด<br>..<br>..<br>..	
15. ท่านยังคงแนะนำให้ผู้ป่วยให้ฉีดวัคซีนป้องกันโรคโควิด-19 และวัคซีนเข็มกระตุ้น อยู่หรือไม่	☐ 1 ให้ (ข้ามไปตอบ ข้อ 16)<br>☐ 2 ไม่ให้
15.1 ถ้าไม่ได้แนะนำ เป็นเพราะเหตุผลใด<br>..<br>..	
16. หากวัคซีนป้องกันโรคโควิด-19 กลายเป็นวัคซีนที่แนะนำให้ต้องฉีดเป็นประจำทุกปี ท่านจะแนะนำให้ผู้ป่วยฉีดวัคซีนเป็นประจำทุกปี หรือไม่	☐ 1 แนะนำ<br>☐ 2 ไม่แนะนำ (ข้ามไปตอบ ข้อ 19)<br>☐ 3 ไม่ทราบ
17. ถ้าท่านให้คำแนะนำ คนกลุ่มใดดังต่อไปนี้ ที่ท่านจะให้ฉีดวัคซีนหรือให้คำแนะนำให้ฉีดวัคซีนป้องกันโรคโควิด-19 (ผู้สัมภาษณ์: ไม่ต้องอ่านออกเสียง)	☐ 1 ผู้ใหญ่ที่มีสุขภาพแข็งแรง<br>☐ 2 ผู้ที่มีอายุตั้งแต่ 65 ปี ขึ้นไป<br>☐ 3 เด็กวัยเรียน

KASETSART UNIVERSITY 1943
COE No.COE66/015
The Kasetsart University
Research Ethics Committee
Date of exemption ...28 November 2023...

	☐ 4 เด็กอายุต่ำกว่า 5 ปี<br>☐ 5 หญิงที่ยังสามารถมีบุตรได้<br>☐ 6 หญิงตั้งครรภ์<br>☐ 7 บุคลากรทางการแพทย์<br>☐ 8 ผู้ติดเชื้อ เอช ไอ วี/ เอดส์<br>☐ 9 ผู้ป่วยโรควัณโรค<br>☐ 10 ผู้ที่มีโรคประจำตัวเรื้อรัง<br>☐ 11 ไม่ทราบ
18. เหตุผลที่สำคัญที่สุด ที่ทำให้ท่าน<u>**ให้คำแนะนำ**</u>วัคซีนป้องกันโรคโควิด-19 คืออะไร (ระบุแล้ว ข้ามไปตอบ ข้อ 20)	....<br>..<br>..
19. เหตุผลที่สำคัญที่สุด ที่ทำให้ท่าน<u>**ไม่ให้คำแนะนำ**</u> วัคซีนป้องกันโรคโควิด-19 คืออะไร	....<br>..<br>..
20. ท่านเคยเผชิญกับความท้าทาย ในขณะที่ท่านกำลังให้คำแนะนำวัคซีนป้องกันโรคโควิด-19 ให้กับผู้ป่วยหรือไม่ โปรดอธิบาย	....<br>..<br>..
20.1 <u>ถ้าเคย</u> ข้ามไป เหตุผลที่ต่อต้านหรือลังเลที่จะรับวัคซีนป้องกันโรคโควิด-19 คืออะไร (ตอบได้มากกว่า 1 ข้อ)	☐ 1 เข้าถึงวัคซีน<br>☐ 1) ความพร้อมของวัคซีน<br>☐ 2) สถานที่ฉีด<br>☐ 3) การเดินทาง<br>☐ 2 เวลา<br>☐ 1) เวลาเดินทางไปคลินิก<br>☐ 2) ระยะเวลารอ (เช่น ต่อคิวฉีดวัคซีน)

KASETSART UNIVERSITY 1943
COE No.COE66/015
The Kasetsart University
Research Ethics Committee
Date of exemption 28 November 2023

	☐ 3 ค่าวัคซีน<br>☐ 4 ค่าบริการ/ค่าใช้จ่ายอื่นๆ<br>☐ 5 ผู้ป่วยกลัวเรื่องความปลอดภัย<br>☐ 6 อื่นๆ (โปรดอธิบาย)<br>..<br>..
21. วัคซีนป้องกันโรคโควิด-19 สามารถป้องกันท่านไม่ให้ป่วยเป็นโรคโควิด-19 ได้ หรือไม่	☐ 1 ได้<br>☐ 2 ไม่ได้<br>☐ 3 ไม่ทราบ

ส่วนที่ 4: ประสบการณ์เกี่ยวกับวัคซีนป้องกันไข้หวัดใหญ่
ผู้สัมภาษณ์: ขอขอบคุณสำหรับการแบ่งปันความคิดเห็นของท่านเกี่ยวกับโรคโควิด-19 ตอนนี้ฉันจะถามคำถามที่เกี่ยวข้องกับประสบการณ์ของคุณกับการฉีดวัคซีนไข้หวัดใหญ่โดยเฉพาะ

ข้อมูลประสบการณ์	ตัวเลือกคำตอบ
0. ท่านได้รับวัคซีนป้องกันไข้หวัดใหญ่ หรือไม่	☐ 1 ได้รับ<br>☐ 2 ไม่ได้รับ
1. กรณีได้รับ หากท่านได้ฉีดวัคซีนป้องกันโรคไข้หวัดใหญ่ การฉีดวัคซีนไข้หวัดใหญ่ส่งผลต่อการตัดสินใจฉีดวัคซีนโควิด-19 หรือไม่	☐ 1 ใช่<br>☐ 2 ไม่ใช่<br>☐ 3 ไม่ทราบ
2. โปรดอธิบายเหตุผลว่า ทำไมส่งผล และทำไมไม่ส่งผล	☐ 1 ส่งผลต่อการตัดสินใจ เหตุผลเพราะ<br>..<br>☐ 2 ไม่ส่งผลต่อการตัดสินใจ เหตุผลเพราะ<br>..
3. จากประสบการณ์ฉีดวัคซีนป้องกันไข้หวัดใหญ่ของท่าน มีผลต่อการตัดสินใจให้คำแนะนำวัคซีนป้องกันโรคโควิด-19 แก่ผู้ป่วย หรือไม่	☐ 1 ใช่<br>☐ 2 ไม่ใช่<br>☐ 3 ไม่ทราบ

COE No.COE66/015
The Kasetsart University
Research Ethics Committee
Date of exemption 28 November 2023

4. โปรดอธิบายเหตุผลว่า ทำไมมีผล และทำไมไม่มีผล	☐ 1 มีผลต่อการตัดสินใจ เหตุผลเพราะ<br>...<br>☐ 2 ไม่มีผลต่อการตัดสินใจ เหตุผลเพราะ<br>...
5. ในปีที่แล้ว ท่านได้รับทั้งวัคซีนป้องกันโรคโควิด-19 และวัคซีนป้องกันไข้หวัดใหญ่ตามฤดูกาล หรือไม่	☐ 1 ใช่ ได้รับทั้งสองอย่าง<br>☐ 2 ไม่ใช่ ไม่ได้รับทั้งสองอย่าง<br>☐ 3 ได้รับวัคซีนป้องกันโรคโควิด-19 เท่านั้น<br>☐ 4 ได้รับวัคซีนป้องกันโรคไข้หวัดใหญ่ตามฤดูกาลเท่านั้น<br>☐ 5 ไม่ทราบ
6. หากวัคซีนป้องกันโรคโควิด-19 และวัคซีนป้องกันโรคไข้หวัดใหญ่ตามฤดูกาล (ฉีดร่วมกัน) ในฤดูกาลไข้หวัดใหญ่นี้ ท่านจะฉีดวัคซีน ทั้ง 2 อย่างนี้ หรือไม่	☐ 1 ใช่ จะฉีดทั้งสองเข็ม<br>☐ 2 ไม่ใช่ จะไม่ฉีดทั้งสองเข็ม<br>☐ 3 ไม่ใช่ จะฉีดเฉพาะวัคซีนไข้หวัดใหญ่ เท่านั้น<br>☐ 4 ไม่ใช่ จะฉีดเฉพาะวัคซีนโควิด-19 เท่านั้น<br>☐ 5 ไม่ทราบ
6.1 ถ้าไม่ฉีด ทำไมถึงไม่ฉีด	....<br>...<br>...<br>...

ส่วนที่ 5 ความคิดเห็น

ความคิดเห็น	คำตอบ
1. ท่านมีข้อคิดเห็นหรือข้อเสนอแนะเกี่ยวกับวัคซีนไข้หวัดใหญ่ สำหรับบุคลากรทางการแพทย์ หรือไม่	....<br>...<br>...

KASETSART UNIVERSITY 1943
COE No.COE66/015
The Kasetsart University
Research Ethics Committee
Date of exemption 28 November 2023

2. ท่านมีข้อคิดเห็นหรือข้อเสนอแนะเกี่ยวกับวัคซีนโควิด-19 สำหรับบุคลากรทางการแพทย์ หรือไม่	....

KASETSART UNIVERSITY 1943
COE No.COE66/015
The Kasetsart University
Research Ethics Committee
Date of exemption ...28 November 2023.........
