# Supplementary material for: Perceptions of influenza and SARS-CoV-2 vaccination among health care personnel in Thailand, 2024
Source: PLoS One. 2025 Aug 14;20(8):e0329473. doi: 10.1371/journal.pone.0329473 (PMC12352830; doi:10.1371/journal.pone.0329473)
Supplement: Attachment — Submitted filename: Comments and responses_R2.docx [file pone.0329473.s005.docx]

3 July 2025

Dear Dr. Sirwan Khalid Ahmed,

Thank you for the opportunity to make additional revisions to improve our manuscript. We have addressed the reviewer’s two comments and provided a point-by-point response to each comment below.

Sincerely,

Prabda Praphasiri, PhD, DrPH
Influenza Division
U.S. Centers for Disease Control and Prevention

Reviewer #2: General Comments
The authors have generally addressed my previous comments appropriately.
However, I would like to offer a couple of follow-up remarks regarding their responses to my earlier comments.
These are detailed below.

Comment 1
Thank you for your response and the revisions made to the manuscript.
Based on your reply, I understand that the open-ended responses were not included in the analysis.
Could you clarify what kind of questions generated these open-ended responses?
Additionally, what was the rationale for excluding them from the analysis?
Even if you judged that analyzing them was not necessary for the purpose of this study, it would be helpful to provide a general overview of the survey content — specifically, the exact questions that were asked in both the multiple-choice and open-ended formats, and which of these were included in the analysis. This information can help readers better understand how the respondents perceived the survey and the potential cognitive or time burden involved in completing it.
I also suggest that you consider briefly stating the reason for excluding the open-ended responses in the revised manuscript. Furthermore, providing a full list of the survey questions — including those not analyzed — as a Supplementary File may help convey the overall scope and structure of the survey more transparently.

Response 1

The open-ended questions provided survey participants the opportunity to provide additional explanation to the multiple-choice questions. We have added a copy of the survey in Thai and English as a supplemental file for readers to review. We have revised the text to provide additional explanation on our decision to not analyze the open-ended response questions in this analysis. On line 134, we explain, “Open-ended responses were used to provide additional clarification to multiple choice responses and were not systematically analyzed.” We also included a reference to the supplemental file on line 104.

Comment 2
Thank you for your explanation regarding how you handled the “refuse to answer” responses.
I agree that such responses might reasonably correspond to low perceived disease severity, vaccine safety, or vaccine effectiveness, as you suggest. However, this assumption may not always hold true in every case. In some cases, it is also possible that the respondent had a high level of perception and chose not to answer intentionally for personal or strongly held reasons.
I also understand that the proportion of such responses was small (<10%) for most perception questions. Nevertheless, given that the perception score serves as a key component in summarizing the survey results in your study, I believe it would be helpful to provide this information for readers’ understanding.
I suggest that you clearly state in the manuscript the rationale for assigning a score of 0 to “refuse to answer” responses. If you agree, it would also be worth briefly acknowledging that this approach may not fully capture the range of possible interpretations for such responses. Finally, I recommend including a note in the manuscript indicating that the proportion of “refuse to answer” responses was low.

Response 2

The response of refusing to answer is indeed ambiguous. We have revised the manuscript as suggested in the limitations paragraph on lines 402-406. We provide some explanation of our rationale and acknowledge the limitations of this approach. This reads, “For perception questions, participants who refused to answer were assigned a score of 0, as we assumed this response corresponded with low perceived disease severity, vaccine safety, or vaccine effectiveness; however, this response is ambiguous, and a score of 0 might not be appropriate in all cases. Overall, the proportion of participants who refused to answer was small (<10%) for most perception questions.”
